# Molecular characterization of vulnibactin biosynthesis in *Vibrio vulnificus* indicates the existence of an alternative siderophore

**DOI:** 10.3389/fmicb.2014.00001

**Published:** 2014-01-24

**Authors:** Wenzhi Tan, Vivek Verma, Kwangjoon Jeong, Soo Young Kim, Che-Hun Jung, Shee Eun Lee, Joon Haeng Rhee

**Affiliations:** ^1^Department of Microbiology, Clinical Vaccine R&D Center, Chonnam National University Medical SchoolGwangju, South Korea; ^2^Department of Chemistry, Chonnam National University College of Natural ScienceGwangju, South Korea; ^3^Department of Pharmacology and Dental Therapeutics, School of Dentistry, Chonnam National UniversityGwangju, South Korea

**Keywords:** *V. vulnifiucus*, siderophore, salicylate, 2, 3-DHBA, hydroxyl radical, AMP ligase

## Abstract

*Vibrio vulnificus* is a halophilic estuarine bacterium that causes fatal septicemia and necrotizing wound infections in humans. Virulent *V. vulnificus* isolates produce a catechol siderophore called vulnibactin, made up of one residue of 2, 3-dihydroxybenzoic acid (2, 3-DHBA) and two residues of salicylic acid (SA). Vulnibactin biosynthetic genes (VV2_0828 to VV2_0844) are clustered at one locus of chromosome 2, expression of which is significantly up-regulated *in vivo*. In the present study, we decipher the biosynthetic network of vulnibactin, focusing specifically on genes around SA and 2, 3-DHBA biosynthetic steps. Deletion mutant of isochorismate pyruvate lyase (VV2_0839) or 2, 3-dihydroxybenzoate-2, 3-dehydrogenase (VV2_0834) showed retarded growth under iron-limited conditions though the latter showed more significant growth defect than the former, suggesting a dominant role of 2, 3-DHBA in the vulnibactin biosynthesis. A double deletion mutant of VV2_0839 and VV2_0834 manifested additional growth defect under iron limitation. Though the growth defect of respective single deletion mutants could be restored by exogenous SA or 2, 3-DHBA, only 2, 3-DHBA could rescue the double mutant when supplied alone. However, double mutant could be rescued with SA only when hydrogen peroxide was supplied exogenously, suggesting a chemical conversion of SA to 2, 3-DHBA. Assembly of two SA and one 2, 3-DHBA into vulnibactin was mediated by two AMP ligase genes (VV2_0836 and VV2_0840). VV2_0836 deletion mutant showed more significant growth defect under iron limitation, suggesting its dominant function. In conclusion, using molecular genetic analytical tools, we confirm that vulnibactin is assembled of both 2, 3-DHBA and SA. However, conversion of SA to 2, 3-DHBA in presence of hydrogen peroxide and growth profile of AMP ligase mutants suggest a plausible existence of yet unidentified alternative siderophore that may be composed solely of 2, 3-DHBA.

## Introduction

*Vibrio vulnificus* is an opportunistic Gram-negative bacterial pathogen that causes fatal septicemia and necrotizing wound infections in susceptible individuals with high serum iron levels (Strom and Paranjpye, 2000). Due to its vital role as a redox cofactor of proteins, iron is an essential micronutrient for most life forms. Since iron availability is limited in biological systems, pathogenic bacteria have evolved an array of intricate mechanisms to scavenge iron from the host (Skaar, 2010). The low molecular weight compound called siderophore binds iron with high affinity (Braun and Killmann, 1999) and is an important virulence factor produced by *V. vulnificus* (Morris et al., 1987; Litwin et al., 1996). Virulent *V. vulnificus* produces a phenolate (catechol) siderophore called vulnibactin that enables it to acquire iron from highly iron saturated host proteins such as transferrin (Stelma et al., 1992).

**Figure 1:**
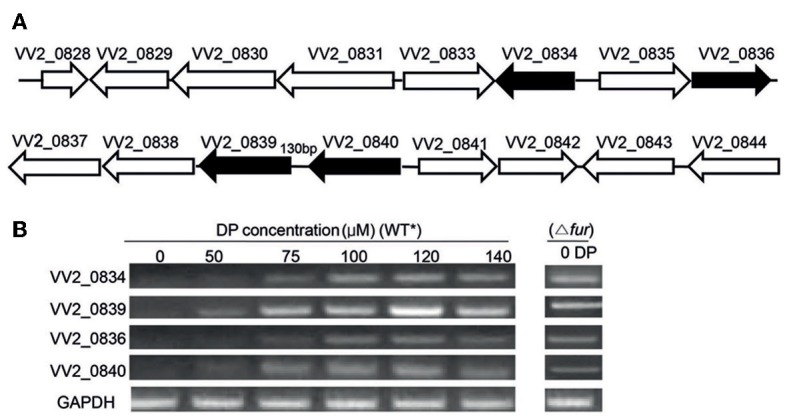
**Gene organization and expression under various iron concentrations. (A)** Organization of Open Reading Frames (ORFs) in the vulnibactin biosynthetic gene cluster located on chromosome 2 of *V. vulnificus*. ORFs filled in black represent the genes of focus in the present study. **(B)** RT-PCR estimated expression of VV2_0834, VV2_0839, VV2_0836, and VV2_0840 in wild type and Fur regulator mutant grown in 2.5% NaCl-HI supplemented with various DP concentrations. ^*^wild type.

Vulnibactin was reported to be composed of one residue of 2, 3-DHBA and two residues of salicylic acid (SA), both of which are involved in the formation of oxazoline rings with L-threonine, which is bound to a norspermidine backbone (Okujo et al., 1994). The chemical structure of vulnibactin is closely related to the *Vibrio cholerae* catechol siderophore, vibriobactin. Vibriobactin was reported to be composed of three residues of 2, 3-DHBA, and like vulnibactin its biosynthesis was also found to be dependent on non-ribosomal peptide synthatases (NRPS) (Griffiths et al., 1984; Keating et al., 2000). Hence, it is highly likely that synthesis of 2, 3-DHBA in *V. vulnificus* is similar to that of *Vibrio cholerae*. The most important precursor in NRPS-dependent siderophore biosynthetic pathway is chorismate. 2, 3-DHBA could be formed from chorismate by a three step reaction catalyzed by isochorismate synthase (Liu et al., 1990), isochorismatase (Rusnak et al., 1990; Litwin et al., 1996), and 2, 3-dihydroxybenzoate-2, 3-dehydrogenase (Liu et al., 1989; Nahlik et al., 1989). Salicylate is a powerful scavenger of highly reactive hydroxyl radicals, resulting in its non-enzymatic conversion to 2, 3-DHBA, 2, 5-DHBA and catechol, with the ratio of these products dependent upon iron concentrations and pH (Hiller et al., 1983; Halliwell et al., 1988; Maskos et al., 1990; Chang et al., 2008). In bacteria, like 2, 3-DHBA, salicylate could also be derived from chorismate. In *Pseudomonas aeruginosa* and *Pseudomonas fluorescens*, isochorismate synthase and an isochorismate pyruvate lyase were identified as being responsible for salicylate biosynthesis (Gaille et al., 2002, 2003). 2, 3-DHBA and salicylate are subsequently activated by AMP ligase as precursors for siderophore assembly (Keating et al., 2000; Khalil and Pawelek, 2011). In *V. vulnificus*, the genes encoding above-mentioned enzymes are located at a single gene cluster in the chromosome 2 (Figure 1A). Previously, by an *in vivo* transcriptome analysis we observed a significantly up-regulated expression of the genes in this cluster (Rhee, unpublished data). In the present study, using molecular genetics tools, we characterize the vulnibactin biosynthetic pathway highlighting the contribution of 2, 3-DHBA and SA to vulnibactin biosynthesis in *V. vulnificus*. We focus on four genes in the mentioned gene cluster (Figure 1A) i.e., VV2_0834 (2, 3-dihydroxybenzoate-2, 3-dehydrogenase), VV2_0839 (isochorismate pyruvate lyase), and two genes encoding putative AMP ligases (VV2_0836 and VV2_0840) for activation of 2, 3-DHBA or SA. By gene deletion and respective in-trans complementation studies we establish the essentiality of these genes in bacterial virulence. Further, by exogenously supplying the products of deleted genes, we found that 2, 3-DHBA is more important than SA for growth under iron limited conditions, and endogenously synthesized SA could serve as a scavenger of hydroxyl radicals, supplying 2, 3-DHBA for siderophore biosynthesis. Furthermore, we cloned and purified VV2_0836 and VV2_0840 encoded AMP ligases and found that both of these enzymes are capable of activating 2, 3-DHBA and SA, and their essentiality for siderophore biosynthesis is dependent on iron levels. By way of molecular dissection of various pathways of siderophore synthesis in *V. vulnificus* we envisage the existence of an alternative siderophore composed solely of 2, 3-DHBA.

## Materials and methods

### Bacterial strains, plasmids, and growth conditions

Bacterial strains and plasmids used in the present study are enlisted in Table 1. *V. vulnificus* CMCP6 is a clinical isolate from a male patient, isolated at the Chonnam National University Hospital, South Korea. *V. vulnificus* CMCP6 was grown in 2.5% NaCl heart infusion (HI) medium while *Escherichia coli* strains were grown in Luria-Bertani (LB) medium supplemented appropriately with antibiotics. Bacteria were grown at 37°C under shaking conditions (200 rpm). Thiosulfate citrate bile salt sucrose agar (TCBS) (Merck, Darmstadt, Germany) was used as the selective medium for *V. vulnificus*. For *E. coli*, antibiotics were used at the following concentrations: ampicilin (Amp) 100 μg/ml, kanamycin (Km) 100 μg/ml, chloramphenicol (Cm) 30 μg/ml, and tetracycline (Tc) 12.5 μg/ml. For *V. vulnificus*, Amp (20 μg/ml), Tc (2 μg/ml) and Cm (2 μg/ml) were used. To assess the growth of bacteria under iron-limited conditions, overnight grown bacterial cultures in HI medium were washed twice with phosphate buffered saline (PBS, pH 7.2) and inoculated into fresh HI broth supplemented with various concentrations of 2, 2-dipyridyl (DP) (Sigma) to a final concentration of 5 × 10^5^ CFU/ml. The optical density at 600 nm (OD_600_) was measured spectrophotometerically (Ultrospec 6300 Pro, Amersham Biosciences) at selected time points.

**Table 1:** **Strains and plasmids used in this study**.

**Strain or plasmid**	**Description**	**Source/references**
**BACTERIAL STRAINS**		
*V. vulnificus* CMCP6	Clinical isolate from a septicemic patient	Kim et al., 2011
Δ20834	CMCP6 with deletion of VV2_0834	This study
Δ20839	CMCP6 with deletion of VV2_0839	This study
Δ20836	CMCP6 with deletion of VV2_0836	This study
Δ20840	CMCP6 with deletion of VV2_0840	This study
Δ20839/20834	CMCP6 with deletion of VV2_0839 and VV2_0834	This study
Δ20836/20840	CMCP6 with deletion of VV2_0836 andVV2_0840	This study
Δfur	CMCP6 with deletion of *fur* gene	This study
*E. coli*		
DH5α	F-*recA1*; restriction negative	ATCC
SY327 λ pir	Δ(*lac pro*) *argE* (Am) *rif nalA recA56* λ pir lysogen; host for π-requiring plasmids	Miller and Mekalanos, 1988
SM10λ pir	*thi thr leu tonA lacY supE recA*::RP4-2-TcR::Mu λ pirlysogen, oriT of RP4, *Km^r^*; Conjugal donor	Miller and Mekalanos, 1988
**PLASMIDS**		
pDM4	A suicide vector with ori R6K *sacB* and *Cm^r^*	Milton et al., 1996
pLAFR3II	pLAFR3 with *bla* instead of cos site	Simon et al., 1983
pRK2013	IncP *Km^r^*Tra Rk2_ repRK2 repE1	Ditta et al., 1980
pTYB12	N-terminal fusion expression vector in which the N terminus of a target protein is a fused intein tag; *Ap^r^*	New England Biolabs, Inc.
c20834	Fragment containing VV2_0834 cloned into pLAFR3II	This study
c20839	Fragment containing VV2_0839 cloned into pLAFR3II	This study
c20836	Fragment containing VV2_0836 cloned into pLAFR3II	This study
c20840	Fragment containing VV2_0840 cloned into pLAFR3II	This study

*Cm^r^, Cm resistance; Tc^r^, Tc resistance; Ap^r^, Ap resistance; Km^r^, Km resistance*.

### Construction of in-frame deletion mutants by homologous recombination method

The chromosomal in-frame deletion mutants of VV2_0834, VV2_0839, VV2_0836, and VV2_0840 were constructed in *V. vulnificus* by allelic exchange method (Miller and Mekalanos, 1988). Primers used for PCR reactions are enlisted in Table 2. As per requirement, PCR primers were synthesized with overhangs recognized by specific restriction enzymes (REs) for ligation into appropriate vectors. Upstream and downstream 1000 base pair (bp) fragments of target genes were amplified separately and converted to 2 kbp fragments by cross over PCR (Horton et al., 1989). Fusion fragments were digested with appropriate REs and subcloned into pDM4 suicide vector. The resulting recombinant vector was transformed into *E. coli* SM10 λ *pir* and subsequently mated into *V. vulnificus* CMCP6 by conjugation. Stable Cm^*R*^ transconjugants were selected on TCBS agar plate containing Cm. Plating of the transconjugants on 2.5% NaCl-HI agar plate containing 10% sucrose was performed to select clones that experienced the second homologous recombination events forcing excision of the vector sequence and leaving only mutated or wild type allele of the genes. Each in-frame deletion mutation was confirmed by PCR with the chromosomal DNA from the respective mutant as template. The resulting mutant strains are enlisted in Table 1.

**Table 2:** **Primers used in the present study**.

**Function and name**	**Nucleotide sequence (5' to 3')**
**CONSTRUCTION OF VV2_0834 DELETION**	
834-UF^*^(SacI)	GACCGAGCTCAGGATGGAAAGGGCTGAT
834-UR^**^	GATAACGTTTTTATGTAATAACTTGCTGAAGCA
834-DF^#^	TTCAGCAAGTTATTACATAAAAACGTTATCTCT
834-DR^##^ (ApaI)	GCGGGCCCGAACCGGCCAGCGGATTG
**CONSTRUCTION OF VV2_0839 DELETION**	
839-UF (SpeI)	GGACTAGTGGCGTTGGTGCCTGCATT
839-UR	CGAGTGATAAGCTACACGGTTATTTCCTTTCG
839-DF	AGGAAATAACCGTGTAGCTTATCACTCGTAAA
839-DR (ApaI)	GCGGGCCCTTCACACTTAGGCGACGG
**CONSTRUCTION OF VV2_0836 DELETION**	
836-UF (SacI)	GCCGAGCTCGCGAATCAGACAATCCGG
836-UR	TACTTTTCGCTACTACATGCCGATACCTTATGC
836-DF	TAAGGTATCGGCATGTAGTAGCGAAAAGTAAGT
836-DR (ApaI)	GTGGGCCCTAACAACGTCAGCTAGGC
**CONSTRUCTION OF VV2_0840 DELETION**	
840-UF (SacI)	GCCGAGCTCCGGGCTGAGCGCTTCAAC
840-UR	GCAAGGATAGATATGTGATGGTTCTACAACCTA
840-DF	GTT GTAGAACCATCACATATCTATCCTTGCCTT
840-DR (ApaI)	GTGGGCCCCACGAGGACCAATGATGA
**COMPLEMENTATION**	
836-F (EcoRI)	CGGAATTCGAACTCTGTACAATCTGA
836-R (SalI)	ACGCGTCGACCTAAGCACTCAAACCAAG
840-F (BamHI)	CGGGATCCATTAAACTGATGGAGTCGAG
840-R (EcoRI)	CGGAATTCTCACGGCAGAATGGTGCTT
**RT-PCR**	
RT-839F	GTGGCATCGTTGAAGAAACC
RT-839R	AATCTTGGCTCACTGGCG
RT-836F	CAATGGGTTTCGCTCGCT
RT-836R	AGCAGCTCGGAGTGATTG
RT-840F	GCAATTTGCCGCTGTGGC
RT-840R	GGCGAATTTCGACCAAACC
RT-834F	AACAGACTCCGGCTGAGAAA
RT-834R	GGCGAAAGGTATTGGTTTCA
**RECOMBINANT PROTEIN CONSTRUCTION**	
836-F (EcoRI)	GGAATTCCATATGAACTCTGTACAATCTGA
836-R (SalI)	ACGCGTCGACCTAAGCACTCAAACCAAGCT
840-F (NdeI)	CGAAGCATATGGTCGTCGCGACAACCG
840-R (EcoRI)	CGGAATTCTCACGGCAGAATGGTGCTT

*Underlined sequences indicate the RE recognition sites with their respective names written in parenthesis*.

* *Up forward*,

** *Up reverse*,

# *Down forward*,

## *Down reverse*.

### Complementation of mutants

For complementation of the mutants, DNA fragments containing wild type genes with respective promoters were amplified using primers listed in Table 2. Amplified DNA fragments were cloned into pCR2.1 TOPO vector (Invitrogen). Fragments containing genes were cut out by appropriate REs and subcloned into the broad host range vector pLAFR3II (Kim et al., 2003). The resulting plasmids were transferred into the mutant strains by the triparental mating using a conjugative helper plasmid pRK2013 (Ditta et al., 1980). The transconjugants were screened on TCBS agar plates containing appropriate antibiotics and confirmed by PCR.

### RNA extraction and reverse transcription (RT-PCR)

Total RNA from log phase *V. vulnificus* was extracted using RNeasy mini kit (QIAGEN, Germany) in accordance with the manufacturers' protocol. The quality of RNA was assessed using the NanoDrop ND-1000 spectrophotometer (Thermo Fisher Scientific, USA). The cDNA (from total RNA; 1 μ g/reaction mixture) was synthesized with the QuantiTect® Reverse Transcription Kit (Qiagen) according to manufacturer's instructions. 0.5 μ L of cDNA was used in each PCR reaction. Primers used for RT-PCR are listed in Table 2. Fragments were resolved by electrophoresis on 1% agarose gel.

### *in vitro* salicylate (SA) measurement

SA produced in culture supernatants was determined as previously described (Meyer et al., 1992; Leeman et al., 1996). In brief, *V. vulnificus* strains were grown in Tris-HCl-buffered minimal medium (100 mM pH 7.5, 1.1 g NH_4_Cl, 0.27 g KH_2_PO_4_, 25 g NaCl, 4 g succinate, 2 g casamino acids/L) at 37°C for 24 h. Cells were removed by centrifugation and culture supernatants were acidified with 1 N HCl to pH 2.0 and SA was extracted into CHCl_3_ by vigorous shaking (culture supernatant: CHCl_3;_ 3:1). For quantitative measurements, 1 volume of 2.5 mM FeCl_3_ was added to the CHCl_3_ phase. The absorbance of purple Fe-SA complex developed in the aqueous phase was measured at 527 nm and quantified against a standard of SA dissolved in the same growth medium.

### Detection of catechol siderophore production

Siderophore production was detected in bacterial cultures grown in 2.5% NaCl-HI broth or CM9 minimal medium (0.4% glucose, 0.2% sodium succinate, 10 mg/L glutamate, 0.1 μM FeCl_3_). Overnight grown bacterial cultures were washed in PBS and subcultured into 2.5% NaCl-HI supplemented with DP, or CM9. Siderophore production was quantified by the Arnow test (Arnow, 1937). Briefly, 0.2 ml of culture supernatant, 0.2 ml of 0.5 N HCl and 0.2 ml NaNO_2_-Na_2_MoO_4_.2H_2_O was mixed. After the formation of yellow color, 0.2 ml of 1 N NaOH was added resulting in the generation of red color. Total volume was brought to 1 ml with distilled water and the absorbance was measured at 510 nm using 2, 3-DHBA dissolved in the growth medium as standard. DP was assessed not to interfere with the Arnow test. Siderophore concentration was normalized to bacterial cell density (μM/OD_600_).

### Production, purification and characterization of AMP ligase recombinant proteins

VV2_0836 and VV2_0840 encoding putative AMP ligases were amplified from *V. vulnificus* CMCP6 genomic DNA and cloned into the plasmid pTYB12 (New England Biolabs, Inc.). Amplified DNA fragments were sequenced by the dideoxy-chain termination method (Macrogen Inc., South Korea). Resulted plasmids were transformed into electrocompetent *E. coli* ER2566 (New England Biolabs, Beverly, MA) using the *E.coli* Pulsar (BioRad Inc.), for protein expression. Proteins were purified from IPTG induced (0.4 mM) transformed *E. coli* ER2566 after sonication (Sonics & Materials Inc. UK, Ltd.), by affinity chromatography using IMPACT™-CN Protein Purification System (New England Biolabs). The purity of recombinant proteins was confirmed by sodium dodecyl sulfate-polyacrylamide gel electrophoresis (SDS-PAGE). The concentration of the obtained proteins was determined by Quick Start™ 1 × Bradford dye (Bio-Rad Laboratories) and purified proteins were stored at −80°C in stock buffer (25 mM Tris, pH 8.0, 10 mM MgCl_2_, 5 mM DTT, and 10% glycerol).

AMP ligase activity of purified recombinant protein was determined by measuring the pyrophosphate release (Rusnak et al., 1989). This reaction was coupled with the pyrophosphatase reaction. Reaction mixtures (total volume: 100 μ L) containing 1 μM of VV2_0836 or VV2_0840 recombinant proteins, 0.2 unit of *E. coli* inorganic pyrophosphatase (Sigma), 75 mM Tris (pH 7.5), 10 mM MgCl_2_, 1.5 mM ATP, 5 mM DTT, and 0.6 mM salicylate (Junsei) or 2, 3-DHBA (Sigma) were incubated at 37°C. The amount of inorganic phosphate (Pi) produced in the reaction mixture was assayed by measuring the chromophore generated after mixing with an ammonium molybdate-malachite green solution at 620 nm (Sousa et al., 2006; Khalil and Pawelek, 2011).

### Determination of LD_50_ in mice

The LD_50_ of mutants to mice was determined by our previously described method (Kim et al., 2008). Briefly, 7-week-old specific pathogen-free (SPF) randomly bred ICR female mice (5 mice/group) were inoculated intraperitoneally with 10-fold serial dilutions of test strains (10^9^–10^5^ cfu/mouse). Deaths were observed for 48 h and LD_50_ values were calculated by the method of Reed and Muench (1938). All animal experiments were approved by the Animal Care and Use Committee of Chonnam National University, South Korea.

### Statistical analysis

Statistical significance of the growth differences at each time point and differences in siderophore production between strains were compared using the Student's *t*-test. *P*-values less than 0.05 were considered statistically significant. Statistical values were calculated using GraphPad Prism 5 or Microsoft Excel, wherever appropriate. All experiments were repeated three times in triplicates, and results from representative experiments are shown.

## Results

### Expression of VV2_0834, VV2_0839, VV2_0836, and VV2_0840 is induced by iron-limitations and is under the control of fur

To investigate the effect of iron levels on the expression of selected target genes, total RNA was extracted from wild type CMCP6 cultured in 2.5% NaCl-HI supplemented with various amounts of DP. Expression of target genes could not be detected in iron sufficient conditions (Figure 1B). However, expression of VV2_0839 could be detected when DP was added to a final concentration of 50 μM, while that of other three genes could be detected at 75 μM DP concentration (Figure 1B). Fur (ferric uptake regulator) is the global regulator of iron acquisition system (Escolar et al., 1999; Panina et al., 2001). To confirm the regulatory effect of Fur, we developed a Fur deletion mutant of *V. vulnificus* and checked the expression of the four selected genes in this mutant grown in 2.5% NaCl-HI without any DP (Figure 1B). The expression of these four genes was found to be upregulated in the mutant suggesting that Fur acted as a repressor for expression of these four genes under iron-rich conditions.

### SA is dispensable while 2, 3-DHBA is essential for *V. vulnificus* growth under iron-limited conditions

**Figure 2:**
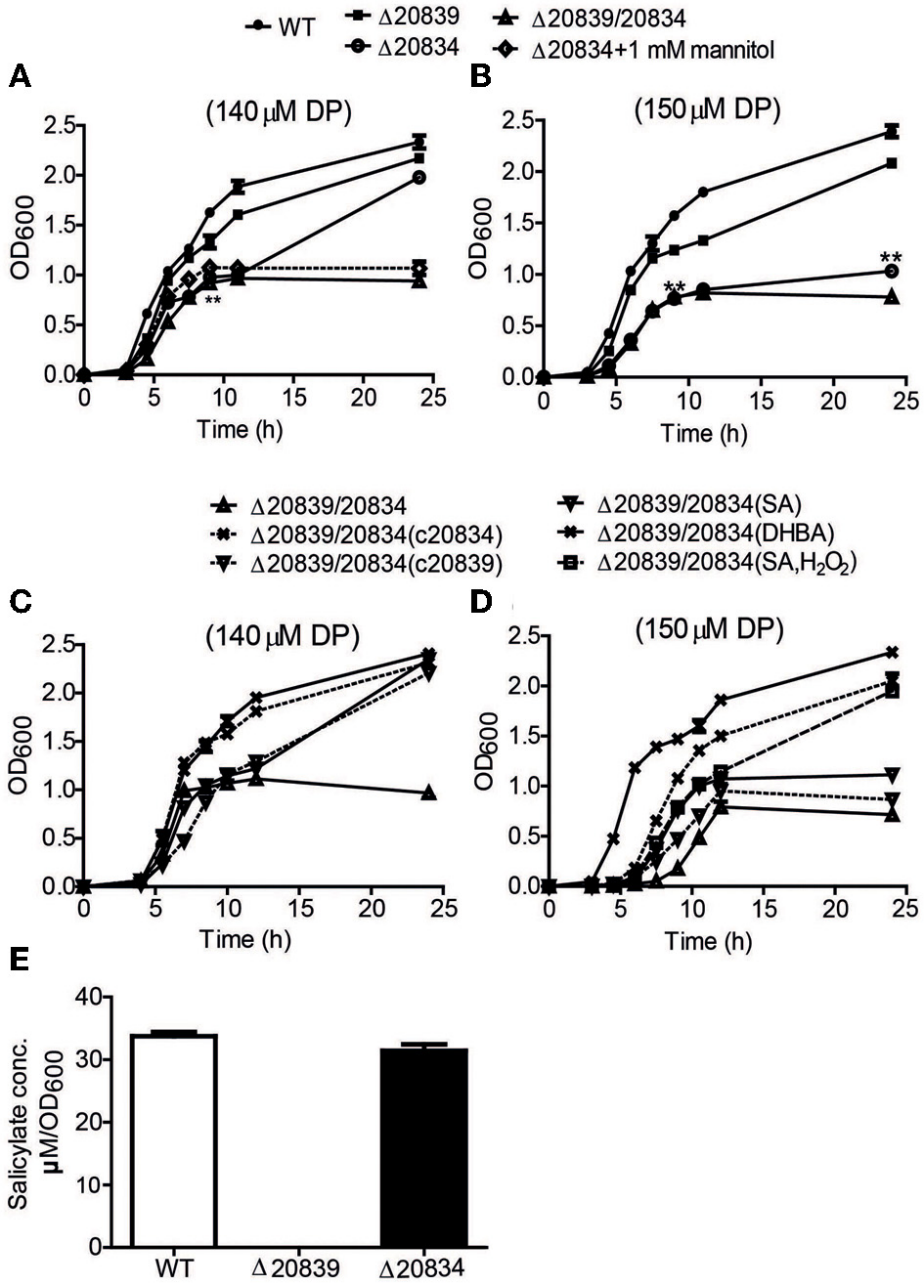
**Growth characteristics and SA production by WT strain and respective mutants.** Bacteria were grown in 2.5% NaCl-HI broth supplemented with either 140 μM DP **(A,C)** or 150 μM DP **(B,D)**. In some growth assays bacterial deletion mutants were complemented with plasmids carrying respective genes. Depending upon experiments mannitol (1 mM), SA (5 μM), DHBA (5 μM), or SA (5 μM) with H_2_O_2_ (10 μM) was added along with DP. SA produced by wild type and mutants cultured in minimal media was also assessed **(E)**. OD_600_ values at each time point are the means of two independent experiments done in triplicate. The error bars represent the standard errors. **P < 0.01.

The siderophore vulnibactin had been reported to contain one residue of 2, 3-DHBA and two residues of SA (Okujo et al., 1994). To investigate the importance of these two substrates for siderophore assembly, mutants with in-frame deletions of respective synthesis genes (VV2_0834 and VV2_0839) were constructed. The growth of mutants was estimated in iron-limited conditions. Against our expectations, Δ20839 mutant showed only a slight growth defect (*P* > 0.05) (Figures 2A,B), despite SA being a major building block of vulnibactin. To confirm the function of the gene VV2_0839, SA production in Δ20839 mutant was measured. No SA production was observed in Δ20839 mutant (Figure 2E).

Under 140 μM DP condition, Δ20834 exhibited initial significantly retarded growth (*P* < 0.01, 9 h time point) followed by an increased growth rate, ultimately catching up with the wild type strain (Figure 2A). However, the same mutant could not catch up the growth if the DP concentration was increased to 150 μM (Figure 2B). The double mutant (Δ20839/20834) manifested more profound growth defect than single gene mutants under both 140 and 150 μM DP conditions. These results clearly suggest that though VV2_0839 is essential for SA synthesis but SA itself is dispensable, while 2, 3-DHBA is essential for the vulnibactin-dependent growth of bacterium under iron-limited conditions. More importantly, these results suggest that vulnibactin, composed of two SA and one 2, 3-DHBA may not to be the only type of siderophore secreted by *V. vulnificus* in response to iron-limitations.

### Catechol siderophore production is impeded by the mutation of VV2_0834 and VV2_0839

**Figure 3:**
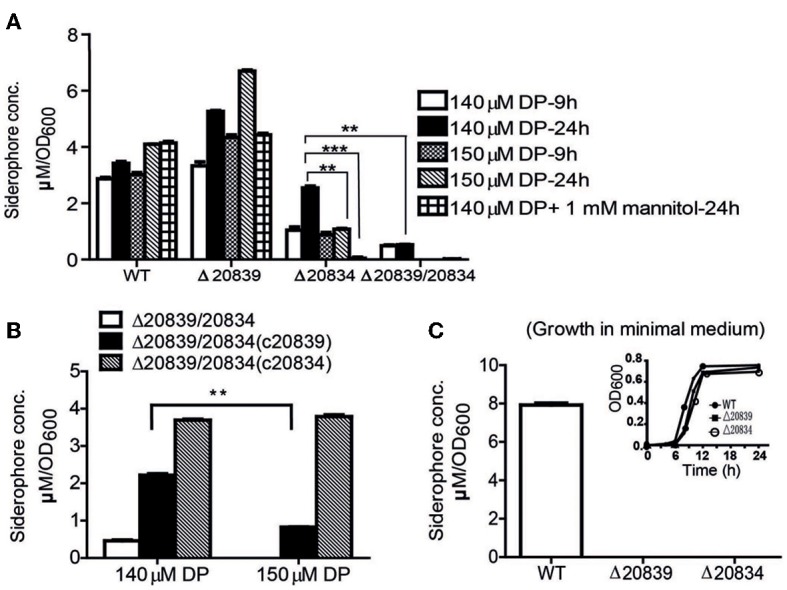
**Siderophore production measurement by Arnow test. (A)** WT and mutants were grown in 2.5% NaCl-HI supplemented with 140 or 150 μM DP for 9 or 24 h. In some experiments mannitol (1 mM) was added along with DP. **(B)** Siderophore levels produced by double mutant and its single gene complemented strains grown in 2.5% NaCl-HI with added DP. **(C)** Siderophore levels produced by WT or single gene mutants grown in minimal medium. Satellite graph represents the growth profile of respective bacterial strains in minimal medium. Siderophore concentration was normalized to cell density: concentration (μM)/OD_600_ of cell culture. Error bars represent standard error of mean (*n* = 3). ^**^P < 0.01, ^***^P < 0.0001.

To further ascertain the importance of genes responsible for biosynthesis of the catechol siderophore in *V. vulnificus*, levels of siderophore production in mutants were measured by the Arnow test (Arnow, 1937). In 2.5% NaCl-HI broth supplemented with 140 or 150 μM DP, more catechol siderophore was secreted by the Δ20839 mutant than the wild type strain (Figure 3A). But in minimal medium, no siderophore was detected in the culture supernatant of Δ20839 (Figure 3C) even though this mutant showed same growth levels as wild type (Figure 3C, satellite) suggesting the presence of an alternate siderophore composed solely of 2, 3-DHBA, produced in iron limited HI broth but not in minimal medium. However, results indicated that the siderophore synthesized without SA was less efficient in acquiring iron since the Δ20839 mutant was slightly impeded compared with wild type despite a higher level of siderophore produced in the culture. Interestingly, we found that Δ20834 mutant was capable of secreting catechol siderophore in 2.5% NaCl-HI supplemented with 140 μM DP, and that siderophore was accumulated in the late growth stage (Figure 3A). The accumulated siderophore in the later growth phase seems to be responsible for the catch-up growth of the mutant (Figure 2A). Though the siderophore amount produced by the single mutant was less than that secreted by wild type, it was significantly higher than that secreted by the double mutant (Δ20839/20834) (Figure 3A). However, the siderophore production in 2.5% NaCl-HI with 150 μM DP and minimal medium was nearly abolished in both single and double mutants (Δ20839/20834) (Figures 3A,C) indicating that somehow SA contributed to the later growth stage production of catechol siderophore in Δ20834 mutant.

### Hydroxyl radicals facilitate the production of 2, 3-DHBA from SA in VV2_0834 mutant (Δ20834)

During stationary growth phase, bacterial cells experience stressful conditions such as decreased pH or increased ROS in culture medium (Storz and Imlay, 1999; Poole, 2012). It has been previously reported that in the presence of free hydroxyl radicals SA is chemically converted to catechol acid, 2, 3-DHBA and 2, 5-DHBA (Grootveld and Halliwell, 1986). Thus, we presumed that SA produced by the Δ20834 mutant could have been attacked by ROS-derived hydroxyl radicals, which consequently lead to the formation of 2, 3-DHBA used for the ultimate production of vulnibactin during the observed late stage catch-up growth (Figure 2A). Mannitol has been shown to be an effective quencher of hydroxyl radicals, inhibiting the production of 2, 3-DHBA from SA (Wendel, 1987). To confirm our hypothesis that SA was being converted to 2, 3-DHBA, we grew the Δ20834 mutant in the presence of various concentrations of mannitol and found that 1 mM mannitol inhibited the late stage growth of Δ20834 mutant (Figure 2A). These results were further corroborated by measuring catechol siderophore production in Δ20834 mutant after mannitol supplementation. Compared to no mannitol condition, siderophore production in the Δ20834 mutant was significantly impeded in the presence of mannitol (Figure 3A). These results conclusively point toward the possibility of SA hydroxylation by free hydroxyl radicals.

### SA or 2, 3-DHBA can restore the defect of Δ20839/20834 double mutant

To further confirm the role SA and 2, 3-DHBA in the vulnibactin biosynthesis, we supplied the double mutant Δ20839/20834 with either SA or 2, 3-DHBA and observed the growth profile in iron-limited 2.5% NaCl-HI. As expected, 2, 3-DHBA fully restored the growth of double mutant in iron-limited conditions regardless of DP concentration (Figures 2C,D). However, SA could rescue the growth of double mutant in iron-limited condition till 140 μM DP concentration showing a growth profile similar to the Δ20834 mutant under 140 μM DP condition (Figures 2A,C). If the concentration of DP was increased to 150 μM, SA alone could not rescue the growth defect of double mutant that could however be rescued in presence of H_2_O_2_ (Figure 2D). Considering that the generation of hydroxyl radical would be dependent upon iron availability and bacterial growth, it is likely that self-generated hydroxyl radicals from the double mutant were not sufficient to hydroxylate SA to 2, 3-DHBA for supporting mutant growth in 150 μM DP condition. Growth patterns similar to that obtained after SA or 2, 3-DHBA supplementation of double mutant (Δ20839/20834) were obtained after complementation with either gene i.e., VV2_0839 or VV2_0834 (Figures 2C,D). Moreover the decrease pattern of siderophore in the presence of DP (Figure 3A) was similar to that observed with respective single deletion mutants (Figure 3B).

### Activity of AMP ligases is affected by iron level

**Figure 4:**
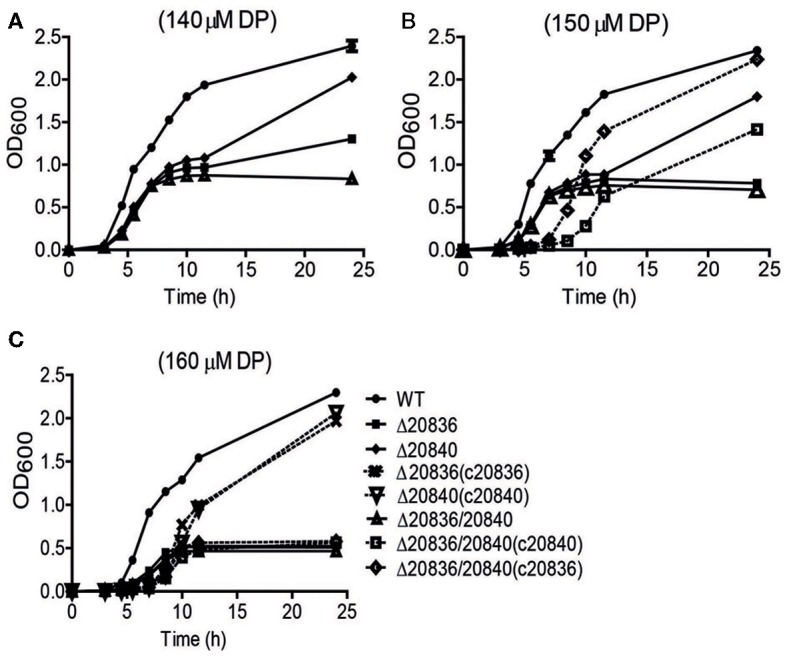
**Growth characteristics of wild-type *V. vulnificus* CMCP6 and various AMP ligase gene mutants under iron-limited conditions.** WT, Δ20836, Δ20840, Δ20836/20840, and complemented strains were grown in 2.5% NaCl-HI broth with added 140 μM **(A)**, 150 μM **(B)**, or 160 μM DP **(C)**. OD_600_ value at each time point represents the mean of two independent cultures of each strain tested, and the error bars represent the standard error of mean.

In the siderophore biosynthetic gene cluster, two genes (VV2_0836 and VV2_0840) encode putative AMP ligases for activation of 2, 3-DHBA and initiating siderophore assembly (Khalil and Pawelek, 2011). Their amino acid sequences showed 42% similarity. To characterize the roles played by these two genes in the siderophore synthesis, in-frame deletion mutants of these genes were constructed and growth was tested under iron-limited conditions. The single gene mutant Δ20840 showed more severe growth defect at only higher DP concentrations while growth of the Δ20836 strain was significantly retarded even at lower concentrations of DP (Figures 4A–C) indicating a more dominant role played by VV2_0836 in vulnibactin assembly. As expected, the growth of double mutant Δ20836/20840 was significantly retarded by iron limitation that nonetheless could be restored fully by complementation with VV2_0836 till at least 150 μM DP condition, while only partially when complemented with VV2_0840 (Figure 4B). In 160 μM DP condition, the growth of double mutant could not be restored by either of the genes while growth defect of single gene mutants could be restored completely by respective gene complementation (Figure 4C). These results clearly indicate the essentiality of two AMP ligases for the siderophore biosynthesis under extremely iron-limited conditions.

### Two AMP ligases can activate 2, 3-DHBA and SA

**Figure 5:**
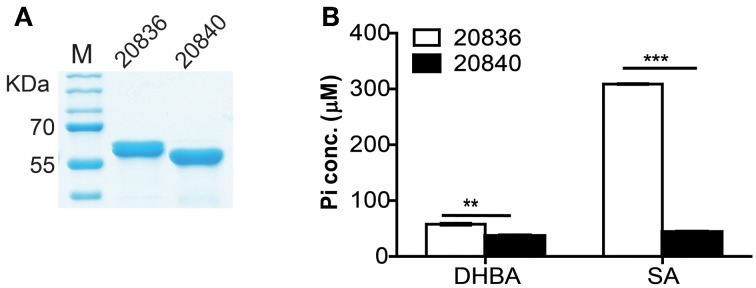
**Purity of the two AMP ligases (A) and their respective activities after 12 min of incubation with 2, 3-DHBA or SA as enzyme substrates (B).** AMP ligase reaction was assayed spectrophotometrically by coupling the formation of PPi (inorganic pyrophosphate) to the pyrophosphatase reactions. The substrate specificity of the two AMP ligases was tested by measuring the formation of inorganic phosphate (Pi). At each time point error bars represent standard errors of mean (*n* = 3). ^**^P < 0.01, ^***^P < 0.0001.

To investigate the substrate specificity of two AMP ligases, we cloned the two genes (VV2_0836 and VV2_0840) into the expression vector pTYB12. Recombinant proteins were purified (Figure 5A) and tested for enzymatic activities with SA or 2, 3-DHBA as substrate. The AMP ligase reaction was assayed spectrophotometrically by coupling the formation of PPi to pyrophosphatase reactions. The generation velocity of Pi indicated the efficiency of AMP ligase. Results indicated that though both AMP ligases could act on 2, 3-DHBA and SA (Figure 5B) but AMP ligase encoded by VV2_0836 had higher enzymatic activity than that by VV2_0840. These results further indicate that SA might be the optimal substrate for AMP ligase encoded by VV2_0836 since its action on SA was much prompt (*P* < 0.01) and significantly higher (*P* < 0.0001) than on 2, 3-DHBA.

### VV2_0839 is the major contributor to mouse lethality

As shown in Table 3, mutation of either of the two AMP ligases (VV2_0836 or VV2_0840) resulted in a marginal 2-fold increase in LD_50_ compared to that of wild type strain. However, as expected, LD_50_ of double mutant Δ20836/20840 increased by 10-fold indicating an important synergistic contribution made by both AMP ligases toward virulence to mice, at least through intraperitoneal route. However, deletion of VV2_0839 or VV2_0834 gene resulted in an increase in LD_50_ by 137 and 2-fold respectively suggesting that SA played a more important role in virulence than 2, 3-DHBA. Considering 10-fold increase in LD_50_ by mutation of both AMP ligases, we could speculate that the significantly impaired virulence of Δ20839 mutant should not solely be due to the defect in the siderophore biosynthesis and SA might play physiological role through other yet unidentified mechanisms warranting the estimation of pathophysiological significance of SA synthesis in future studies.

**Table 3:** **Effect of the mutation on the lethality to mice**.

**Strain**	**Intraperitoneal LD_50_ (CFU/mouse)**	**Fold increase**
Wild type	4.0 × 10^5^	1.0
Δ20834	8.7 × 10^5^	2.1
Δ20839	5.5 × 10^7^	137
Δ20839/20834	5.5 × 10^7^	137
Δ20836	7.4 × 10^5^	1.85
Δ20840	8.9 × 10^5^	2.22
Δ20836/20840	4.0 × 10^6^	10

## Discussion

The importance of iron for pathogenicity of *V. vulnificus* has been demonstrated both clinically and experimentally (Wright et al., 1981; Gulig et al., 2005). Siderophores mediate efficient iron uptake in most bacteria. It was reported that *V. vulnificus* produces a catechol-like siderophore called vulnibactin (Simpson and Oliver, 1983). Genes supposed to be involved in vulnibactin biosynthesis are clustered at a locus of chromosome 2 of *V. vulnificus*. We analyzed the transcriptome of this bacterium using rat peritoneal cavity infection model (Rhee, unpublished study) and found that genes in the aforementioned cluster were highly up-regulated *in vivo*. In the present study, the RT-PCR results showed that iron concentration tightly regulated the expression of selected target genes (VV2_0834, VV2_0839, VV2_0836, and VV2_0840) in this gene cluster. Expression of these genes was not detected in iron-sufficient conditions and could only be found after iron depletion by DP. Expression of each gene was initiated at different DP concentration. Fur is the global regulator of iron acquisition systems (Escolar et al., 1999). Up regulation of these four genes upon deletion of Fur suggested the repressor function of the regulator. However, we also observed expression of these genes in iron rich 0.9% NaCl-HI (data not shown); suggesting that Fur might not be the only regulator of these four genes as these might also be influenced by osmolarity sensing system. In this regard, siderophore genes may also be under the regulation of other global regulators *in vivo*.

In this study, we showed that deletion of VV2_0839 abolished SA production and by extension siderophore production in minimal medium, but in iron-limited nutritious conditions higher amount of siderophore was produced suggesting that SA is dispensable for at least growth of bacterium in nutrition rich conditions as the mutant Δ20839 exhibited insignificant growth defect in HI medium. The chemical structure of vulnibactin was identified to contain one residue of 2, 3-DHBA and two residues of SA and was found to be closely related to that of vibriobactin, a type of catechol siderophore secreted by *V. cholerae* (Griffiths et al., 1984). It is likely that there is an alternative type of siderophore produced by *V. vulnificus*, composed solely of 2, 3-DHBA. However, the iron-sequestering activity of the alternative siderophore seemed to be weaker than vulnibactin (Figures 2A,B). Though for better conclusion of these observations future investigations regarding exact extraction and chemical characterization of siderophores would be required.

We found that the growth characteristics of Δ20834 mutant were tightly associated with the concentration of supplemented DP, hence the available iron levels. In HI with 140 μM or lower DP concentrations, growth of Δ20834 mutant exhibited a stepwise pattern (Figure 2A) having a retarded early growth stage, short stable stage and the late growth stage catching up ultimately with the wild type. The late growth stage of Δ20834 mutant could be inhibited by increasing the DP concentration to 150 μM, by addition of hydroxyl radical scavenger mannitol, or by additional deletion of VV2_0839. SA itself is capable of scavenging reactive hydroxyl radicals (·OH) (Wendel, 1987) leading to the generation of catechol acid, 2, 3-DHBA and 2, 5-DHBA (Grootveld and Halliwell, 1986). In bacterial cultures, hydroxyl radicals could be generated from bacterial respiration or from Fenton reaction. We observed that the pH of culture medium of the Δ20834 mutant fell from 7 to 6 after 9 h of growth in 140 μM DP condition. Hydroxylation of SA is favored in acidic pH resulting in higher conversion rates of SA to 2, 3-DHBA (Chakinala et al., 2007), explaining the stepwise growth pattern of Δ20834 mutant and ultimately reaching the growth profile of the wild type. From these data, we could conclude that SA was used as a scavenger of hydroxyl radicals protecting the bacteria from reactive oxygen species, and also supplying 2, 3-DHBA for siderophore biosynthesis. The complementation results and growth restoration by 2, 3-DHBA, SA, or SA with H_2_O_2_ further substantiated the hypothesis that SA was being converted to 2, 3-DHBA leading to the formation of vulnibactin even in the absence of VV2_0834. These results also emphasized the essentiality of 2, 3-DHBA for bacterial growth in iron-limited conditions.

In the siderophore biosynthetic gene cluster, we found two genes encoding putative AMP ligases. Previously it has been predicted that the residues in the carboxyl acid binding pocket of AMP ligase help in discriminating between 2, 3-DHBA and SA (May et al., 2002). By alignment, the AMP ligase encoded by VV2_0836 was more likely to act on 2, 3-DHBA, while the AMP ligase encoded by VV2_0840 was more likely to act on SA. However, our *in vitro* enzymatic activity test results showed that both of AMP ligases were capable of acting on 2, 3-DHBA and SA. Moreover, AMP ligase encoded by VV2_0836 exhibited much higher activity on SA than AMP ligase encoded by VV2_0840. Both AMP ligases showed relatively low activity on 2, 3-DHBA compared to SA. By measuring the amount of secreted SA and 2, 3-DHBA by the double AMP ligase mutant cultured in the minimal media, we found more SA was accumulated than 2, 3-DHBA (data not shown). It is likely that *V. vulnificus* divide more chorismate for SA production than 2, 3-DHBA. For the growth under iron-limited conditions, VV2_0836 encoded AMP ligase appeared to be more important than VV2_0840 AMP ligase, except that both are required for growth in extremely iron-limited conditions. However, the mechanism through which iron influences the essentiality of AMP ligases remains unclear.

To assess the role of the four genes in the virulence of *V. vulnificus*, we determined the LD_50_ of mutants to ICR female mice. LD_50_ of Δ20839 was significantly higher than Δ20834. Our transcriptome analysis showed that *in vivo* expression of gene VV2_0839 was up-regulated 187-fold compared with *in vitro* culture, while 96-fold for VV2_0834, suggesting a more important role of SA in pathogenicity of *V. vulnificus* than 2, 3-DHBA. In this study, we observed that SA was scavenging the hydroxyl ions *in vitro*, and was capable of supplying 2, 3-DHBA for siderophore biosynthesis. Thus, it seems possible that *V. vulnificus* secrets SA for not only siderophore synthesis but also for protecting the bacterium from damage caused by hydroxyl radicals generated *in vivo*. Another interesting possibility for *in vivo* SA synthesis comes from the fact that SA possesses anti-inflammatory properties. There are a number of reports showing that SA interferes with intracellular signaling pathways such as protein-kinases (MAPK) cascade (Wang and Brecher, 1999) and NF-kB pathway (Kopp and Ghosh, 1994) and may diminish the host inflammatory response to the pathogen. However, this possibility remains to be verified by in appropriate animal model studies.

**Figure 6:**
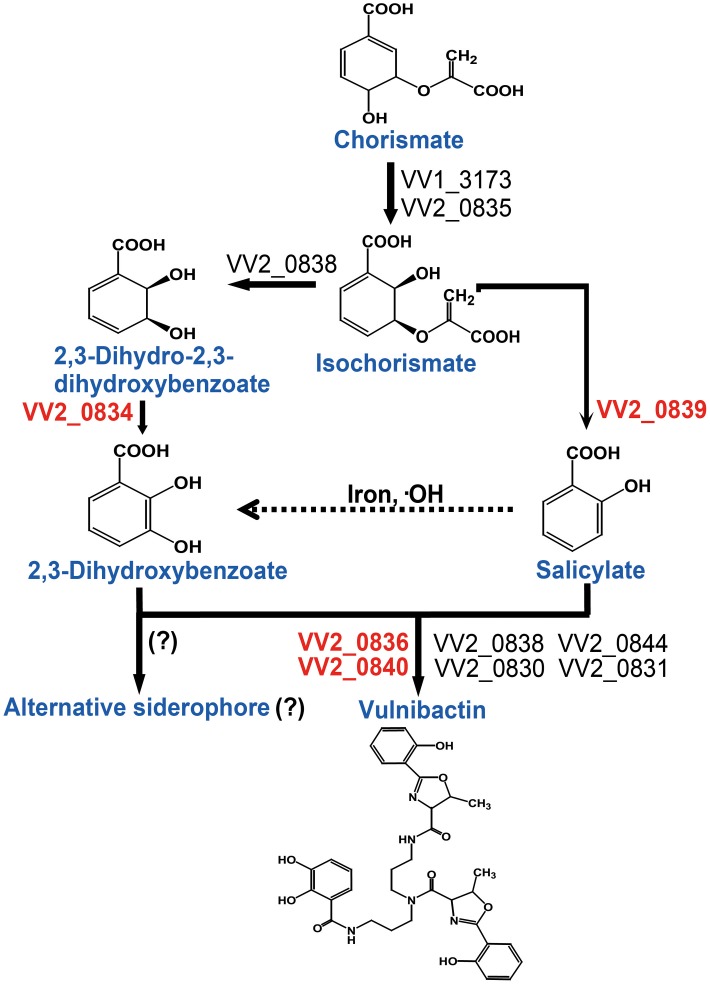
**Proposed vulnibactin biosynthesis pathway in *V. vulnificus*.** 2, 3-DHBA and SA are synthesized from the common precursor chorismate and are activated by AMP ligases encoded by VV2_0836 and VV2_0840 for siderophore assembly. In presence of iron and hydroxyl radicals SA can be non-enzymatically converted to 2, 3-DHBA (dashed arrow) that then may be routed to synthesis of a yet unidentified siderophore composed solely of 2, 3-DHBA. Question marks (?) represent the fact that identity of the reaction or the alternate siderophore is yet to be established.

Taken together, in this study we addressed the important role of 2, 3-DHBA, SA and two AMP ligases for siderophore biosynthesis. Based upon the data obtained from the present study, we constructed a vulnibactin biosynthesis pathway map that is slightly different from that has been predicted in the KEGG database (http://www.genome.jp/kegg-bin/show_pathway?vvu01053+VV2_0834). According to our proposed vulnibactin biosynthetic pathway (Figure 6) isochorismate may be routed to 2, 3-DHBA or SA synthesis, mediated by specific enzymes (VV_20834 and VV_20839 respectively). Synthesized 2, 3-DHBA and SA are assembled into vulnibactin by two AMP ligases (VV2_0836 and VV2_0840). Moreover, SA in presence of hydroxyl radicals may be non-enzymatically converted to 2, 3-DHBA that may be utilized for the production of yet unidentified alternative siderophore that, like vibriobactin, may be composed solely of 2, 3-DHBA. Hence, it is intriguing to suggest that the type and quantity of siderophore synthesized would be determined by the environmental cues sensed by this opportunistic pathogen. We further suggest that SA in *V. vulnificus* serves as a scavenger of hydroxyl radicals and plays an importance role in the virulence of *V. vulnificus*. However, there are several unanswered questions such as exact contribution of SA and vulnibactin, not only as a virulence factor but also as a bacterial defense mechanism to evade host innate inflammatory responses, besides contribution of *in vivo* activated global regulators in siderophore synthesis that needs further exploration.

### Conflict of interest statement

The authors declare that the research was conducted in the absence of any commercial or financial relationships that could be construed as a potential conflict of interest.
